# The Antiapoptotic Function of miR-96 in Prostate Cancer by Inhibition of FOXO1

**DOI:** 10.1371/journal.pone.0080807

**Published:** 2013-11-19

**Authors:** Annika Fendler, Monika Jung, Carsten Stephan, Andreas Erbersdobler, Klaus Jung, George M. Yousef

**Affiliations:** 1 Department of Urology, Charité – University Hospital, Berlin, Germany; 2 Berlin Institute of Urologic Research, Berlin, Germany; 3 Department of Pathology, University Hospital Rostock, Rostock, Germany; 4 Department of Laboratory Medicine, and the Keenan Research Centre in the Li Ka Shing Knowledge Institute, St. Michael’s Hospital, Toronto, Canada; 5 Department of Laboratory Medicine and Pathobiology, University of Toronto, Toronto, Canada; The Chinese University of Hong Kong, Hong Kong

## Abstract

microRNAs (miRNAs) are small molecules that regulate gene expression posttranscriptionally. In a previous study, we identified miR-96 to be upregulated in prostate cancer specimens in comparison to normal adjacent tissue and to be an independent marker of biochemical relapse in a multivariate prediction model. Therefore, we investigated the functional role of miR-96 in prostate carcinogenesis. LNCaP and DU145 prostate cancer cells were transiently transfected with miR-96 precursors and phenotypic changes were analyzed. The miR-96 increased proliferation and impaired apoptosis induced by camptothecine in these cells. In silico target prediction analysis identified FOXO1 as potential pro-apoptotic miR-96 target. miR-96 was able to bind to both bindings sites in the FOXO1 3’ UTR in a luciferase reporter gene assay. Overexpression of miR-96 in LNCaP cells resulted in a reduced FOXO1 expression. Overexpression of FOXO1 induced a strong apoptotic phenotype that was partially rescued by coexpression of miR-96. RT-qPCR and immunohistochemistry of 69 prostate cancer specimens revealed a downregulation of FOXO1 and an inverse correlation of miR-96 and FOXO1 protein expression. In conclusion, we show that miR-96 can regulate apoptosis in prostate cancer, by inhibiting the FOXO1 transcription factor.

## Introduction

MicroRNAs (miRNAs) are a new class of small non-coding RNAs and regulate gene expression posttranscriptionally through inhibition of translation but also through degradation of the corresponding mRNA. Approximately 30% of all mRNAs are predicted to be targeted by miRNAs [1]. Depending on their targets, miRNAs function as tumorsupressors or oncogenes by controlling major pathways of carcinogenesis, including cell proliferation, apoptosis and cell motility [2]. 

Prostate cancer (PCa) is the most common malignant cancer in men and the second leading cause of cancer in the western world [3]. Mechanisms of PCa tumorigenesis are still not fully elucidated and there is a lack of diagnostic and prognostic markers. 

The deregulation of miRNAs in PCa has been proven by numerous studies [4–9]. Their expression correlates with tumor stage and aggressiveness [10]. We have previously identified miR-96 in a set of deregulated miRNAs in PCa. Its expression is correlated with Gleason score and it is an independent marker of biochemical relapse [6]. 

In silico target prediction using miRecords identified FOXO1 among others as a putative target of miR-96. FOXO1 is a member of the forkhead box transcription factors. It exerts its tumorsuppressive function via regulating transcription of important regulators of cell cycle and apoptosis [11,12]. FOXO1 transcriptional activity is regulated via the PI3K/AKT pathway [13]. In breast and endometrial cancers, as well as Hodgkin lymphomas FOXO1 has previously been shown to be regulated by miR-96 [14–16].

We hypothesized that miR-96 may also have an oncogenic function in PCa. To prove this assumption, we (a) studied the influence of miR-96 expression on fundamental cellular characteristics such as proliferation, apoptosis and migration on PCa cell lines, (b) performed in silico target prediction, (c) studied binding of miR-96 to predicted binding sites in the FOXO1 3’ UTR and subsequent changes at the mRNA and protein levels, (d) studied the rescue of FOXO1-induced apoptosis by miR-96 and (e) correlated miR-96 expression with FOXO1 transcript and protein expression in human PCa and matched normal adjacent tissue. We show that miR-96 inhibits camptothecin-induced apoptosis and regulates FOXO1 expression by binding to the 3’-UTR. In PCa specimens, we identified a negative correlation of FOXO1 protein levels and miR-96 expression.

## Materials and Methods

### Tissues and cell lines

Paired normal and malignant tissue samples of 69 PCa patients were collected after radical prostatectomy between 2001 and 2005 at the Charité University Hospital. For each patient clinico-pathological data were collected (Table 1). The study termed “microRNAs as diagnostic and prognostic signatures in urological tumors” (EA1/153/07) was approved by the ethical board of the Charite University Hospital and written informed consent has been obtained.

**Table 1:** Tumor characteristics and clinico-pathological data of patient cohorts.

		**Patients for RT-qPCR (N=69)**		**Patients for TMA analysis (N=64)**	
		**N(%)**		**N(%)**	
Age, years	Median		63		63
	Range		50-72		46-74
Pre-operative PSA^a^, ng/ml	Median		7.07		6.24
	Range		1.71-41.9		2.00-26.0
T stage^a^	pT2a	2 (3)		1 (2)	
	pT2b	11 (16)		3 (5)	
	pT2c	30 (43)		39 (61)	
	pT3a	20 (29)		18 (28)	
	pT3b	6 (9)		3 (5)	
N stage^a^	pN0/pNx	67 (97)			
	pN1	2 (3)			
M stage^a^	M0/Mx	69 (100)			
	M1	0(0)			
Surgical margins	R0	41 (60)			
	R1	27 (39)			
	Rx	1 (1)			
Gleason score	5	3 (4)		1 (2)	
	6	20 (29)		24 (37)	
	7	28 (41)		27 (42)	
	8	11 (16)		8 (12)	
	9	7 (10)		3 (5)	
	10	0 (0)		1 (2)	
Follow-up, month	Median		50.00		35.63
	Range		1-93		0-86
Biochemical recurrence^a^		10 (15)		12 (19)	

a Biochemical relapse was defined as the first postoperative PSA of greater than 0.1 ng/ml, as confirmed by at least 1 subsequent increasing value (persistent PSA increase) after achieving undetectable PSA postoperatively, defined as a detection limit of less than 0.04 ng/ml.

RT-qPCR, real time quantitative PCR, TMA, tissue microarray, PSA, prostate specific antigen, T stage, tumor stage, N stage, lymph node metastases stage, M stage, metastases stage.

Tissues were snap frozen directly after surgery and samples as previously described [6]. Briefly, areas of tumor and normal tissue were identified by haematoxilin-eosin staining by a pathologist and punch-biopsied with a tissue-micro array needle. Only cores with at least 90% tumor content were considered for further analysis.

LNCaP, DU-145, PC3 and 22rv-1 cells were grown in RPMI 1640 (Invitrogen, Carlsbad, CA,) supplement with 10% fetal calf serum and penicillin/streptomycin. Cells were grown in an incubator at 37°C in 5% CO2 atmosphere. BPH-1 cells were grown in RPMI 1640 supplemented with 20% fetal calf serum, 20 ng/ml DHT, 5 µg/ml transferrin, 5 ng/µl sodium selenite and 5 ng/µl insulin. Cell lines were purchased from the American Type Culture Collection (ATCC) or the German Collection of Microorganisms and Cell Cultures (DSMZ). Cell lines are periodically monitored for mycoplasmic contamination by RT-qPCR according to van Kuppeveld et al [17]. Additionally, the identity of prostate cancer cells was verified by the German Prostate Cancer Consortium.

### Tissue Microarray

Formalin-fixed, paraffin-embedded tissue of 64 patients was used for construction of a tissue microarray as described previously [18]. Haematoxilin-eosin staining was performed to identify malignant and benign areas. Areas of interest were punch-biopsied with a 1.5 mm tissue microarray needle and transferred to the recipient block. Each tumor was represented by one core. Normal tissue from colon, pancreas, kidney and 2 prostates and connective tissue was used as control.

### RNA isolation

Total RNA from fresh frozen tissue and cell cultures was isolated using the miRNeasy Minikit (Qiagen, Hilden, Germany) according to the manufacturers instruction as described previously in detail [6].

For extraction of total RNA from cell culture, cells were seeded into 6-well plates in a final concentration of 3 x 10^5^ cells per well and were transfected as described below. After 24 hrs, 1 x 10^6^ cells were harvested in Qiazol (Qiagen). 

RNA yield and A260/280 ratio were monitored with a Nano Drop ND-100 spectrometer (NanoDrop Technologies, Wilmington, DE) and RNA Integrity Numbers (RIN) were assessed with a 2100 Bioanalyzer (Agilent Technologies, Santa Clara, CA). Only RNA samples with a RIN > 6 were included in the analyses.

### Real time quantitative PCR

mRNA levels were analyzed by RT-qPCR. RNA from cells was reverse transcribed using the Omniscript Reverse transcriptase and Oligo-dT primers (Qiagen) according to the manufacturers recommendations. Reactions were performed in a total volume of 20 µl using 1 µg total RNA. Reactions were incubated with a Step One thermal cycler (Applied Biosystems, Foster City, CA). Quantification of FOXO1 from cell lines was performed using the 2 x Fast SYBRgreen Mastermix (Applied Biosystems) using 1 µl cDNA in 20 µl total volume. Primer sequences are listed in table S1. Primers were designed to produce an amplicon spanning at least 1 intron. Specificity of amplification was ensured by primer blasting and melting curve analyses.

mRNA from tissue samples was reverse transcribed using the Transcriptor First Strand cDNA Synthesis Kit (Roche, Mannheim, Germany) from 1 µg RNA in 20 µl total volume. Quantification of FOXO1 was performed using UPL probe #11 (Roche) and Universal Probes Master Mastermix (Roche) in 10 µl total volume.

miRNAs were detected by RT-qPCR using the TaqMan miRNA Assay as previously described [6].

All samples were run in triplicate and mean value and SD were calculated. FOXO1 expression was normalized to TUBA1B [19], while miR-96 was normalized to miR-130b [6]. Standard curves were measured for each gene to correct for efficiency (FOXO1: E=1.95; TUBA1B: E=1.96; miR-96: E=1.83; miR-130b: E=1.83). Normalization was performed as previously described [6].

### Construction of luciferase vectors

Target gene 3’ UTR sequences were cloned into the pMiR report vector (Ambion Austin TX, USA). Target sequences of the FOXO1 3’ UTR were amplified from BPH1 cDNA using AmpliTaq Gold DNA Polymerase (Invitrogen, Carlsbad, CA) and gene-specific primers (table S1), cloned into the pCR 2.1-TOPO vector (Invitrogen) and amplified in TOP10 chemically competent cells (Invitrogen). Plasmid DNA from positive clones was extracted with the QIAprep Spin Miniprep Kit (Qiagen) and sent to sequencing using M13 uni (-21) and M13 rev (-29) primers (Eurofins MWG GmbH, Ebersberg, Germany). Restriction digestions of positive pCR2.1 vectors and the pMiR report vectors were performed with SacI and SpeI (New England Biolabs, Ipswich, MA). Restricted inserts and pMiR report vector were ligated using 1 U T4 DNA-Ligase (Invitrogen) and amplified in chemically competent DH5α cells (Invitrogen). Positive clones were identified by colony PCR. Plasmid DNA was isolated using the QIAprep Spin Miniprep Kit (Qiagen).

### Transfection of PCa cell lines with pre-miRNAs, miRNA inhibitors and plasmid DNA

LNCaP and DU145 cells were transfected with pre-miRNA precursors, anti-miRNA inhibitors or pre-miR-NC#1 (Applied Biosystems) in a final concentration of 10 nM or 500 ng FOXO1 full length clone (Origene, Rockville MD, USA) using siPort NeoFX transfection agent (Applied Biosystems). For each assay, a no transfection control was carried along. For luciferase reporter gene assays cells were additionally transfected with 500 ng pMiR report vector and β-galactosidase control vector.

### Luciferase reporter gene assay

LNCaP cells were grown to 80% confluency and seeded into 6-well plates at a final density of 2 x 10^5^ cells per well. Forty-eight hours post transfection cells were detached with a scraper and total protein was extracted in 80 µl lysis buffer (Applied Biosystems). Luciferase and β-galactosidase activity in the extracts was detected using the Dual-Light Combined Reporter Gene Assay System (Applied Biosystems) according to the manufacturer’s instructions. Luciferase expression was normalized to β-galactosidase expression. Each reaction was performed in duplicate and results are shown as average of three independent assays.

### Western Blot

For extraction of total protein from cell culture, cells were seeded into 6-well plates in a final concentration of 3 x 10^5^ cells per well and were transfected as described above. Protein was extracted after 72 hours in 100 µl NENT buffer (20 mM TRIS-HCl, 50 mM NaCl, 1 mM EDTA, 2% NP40, 0.2% SDS, pH 7.5) or RIPA buffer (150 mM NaCl, 1% NP40, 0.5% sodium deoxycholate, 0.1% SDS, 50 mM tris base, 100 µM PMSF, 1 µg/ml aprotinin, 10 µg/ml soybean trypsin inhibitor, 2 mM EDTA). Total protein was resolved on 10% SDS-PAGE gels and transferred to a 0.45 µm polyvinyldifluoride membrane. The membran was blocked by 2% skimmed milk and probed with the following antibodies: rabbit anti-FOXO1 pAb, mouse anti-ACTB mAB (Sigma Aldrich, Munich, Germany), rabbit anti-Akt pAb, rabbit anti-pAkt pAB (Cell Signaling Technologies, Danvers, MA), and goat anti-rabbit, HRP conjugated or rabbit anti-mouse, HRP-conjugated secondary Ab (DAKO, Hamburg, Germany). Membranes were stained with ECL (Pierce, Bonn, Germany) or advanced ECL solution (GE Healthcare, Munich, Germany) for 5 min and developed on the Fluoro-S multiimager (BioRad, Munich, Germany). To determine relative protein concentration, band intensity was calculated with ImageJ 1.43r (http://rsb.info.nih.gov/ij) and normalized to β-actin.

### Proliferation assay and analysis of cell cycle

Proliferation of pre-miR-96 transfected LNCaP cells was assessed by metabolic conversion of 3-[4,5-dimethylthiazol-2-yl]-2,5-diphenyl tetrazolium bromid (MTT) (Sigma Aldrich). Cells were grown to 80% confluence and seeded into 96-well plates at a final concentration of 6 x 10^3^ cells per well and transfected as described above. Cells were allowed to rest for 24 hrs and were subsequently serum-starved. Cultures were incubated in 5 mg/ml MTT for 4 h and lysed in 10% SDS in 0.01 M HCl. Lysates were incubated at 37 °C overnight and absorbance of formazan was measured at 550 nm. Each reaction was performed in triplicate and results are shown as average of three independent assays. For cell cycle analysis cells were grown to 80% confluence and seeded into 6-well plates at a final concentration of 1.5 x 10^5^ cells per well and transfected as described above. Cells were allowed to rest for 24 hrs and were subsequently serum-starved for 24 hrs. Cells were stained with 20 µg/ml propidium iodide supplemented with 200 µg/ml RNAse in 0.1% Triton X-100 and analyzed using the FacsScan flow cytometer and the CellQuest Software (BD Biosciences, Mississauga, ON, Canada). Each sample was measured in duplicate and results are presented as average of three independent assays.

### Wound-healing assay

To asses the migrative phenotyp of DU-145 cells upon miR-96 transfection we performed a wound-healing assay. Cells were grown to 80% confluence, seeded into 6-well plates and transfected as described above. Cells were allowed to grow to confluence and monolayer was scratched with a 100 µl pipette tip. Remigration of cells to the wound was assessed by life cell imaging for 24 h under serum starvation. Percentage of open area was measured with the software TScratch software [20] at defined time points (0,5,10,15,20 h). All values were normalized to the percentage of open area at 0 h. Two randomly chosen areas of each scratch were analyzed. Data are represented as average of three independent assays.

### Apoptosis assay

LNCaP and DU145 cells were grown to 80% confluence and seeded into 6-well plates at a final concentration of 2 x 10^5^ cells per well and transfected as described above. Apoptosis was induced with 10 µM camptothecin (CPT) (Sigma Aldrich) in serum free media for 24 h. Cells were stained with Annexin V-FITC (Invitrogen) and propidium iodide (PI) (Invitrogen) Cultures were analyzed using the FacsScan flow cytometer and the CellQuest Software (BD Biosciences, Mississauga, ON, Canada). Each sample was measured in duplicate and results are presented as average of three independent assays.

### Immunohistochemistry

Immunohistochemistry was performed on 2 µm slides of the tissue microarray as described previously [21]. In brief, tissue was deparaffinized in xylol and antigens were demasked in a pressur cooker. Slides were probed with rabbit anti-FOXO1 mAb (Cell Signaling Technologies), biotinylated linker-Ab and streptavidin-conjugated secondary Ab (DAKO) and stained with Fast Red to the desired intensity. Nuclei were counterstained with haemalaun. Slides were covered using Aquatex mounting medium (Merck KgH, Darmstadt, Germany). Staining of FOXO1 was analyzed in tumor glands and normal adjacent tissue for each patient if applicable. Since overall staining for FOXO1 was only weak, it was scored as present (1) or absent (0) only.

### In silico target research

In silico target search for miR-96 targets was performed using miRecords (http://mirecords.biolead.org/). We used the criterion that a putative target had to be detected by TargetScan, miRanda, PicTar and two other prediction algorithms. Location of miR-96 binding sites in the 3’ UTR of FOXO1 were analyzed using TargetScan 5.1.

### Statistical analyses

Statistical analyses were performed with GraphPad Prism version 5.01 (GraphPad Software, San Diego, CA), MedCalc version 10.3.2 (MedCalc Software, Mariakerke, Belgium) or SPSS 19.0 (IBM, Somers, NY). Student’s t-test, one-way or two-way ANOVA, Kolmogorov-Smirnof normality test, Wilcoxon signed rank test, McNemar test, Kaplan-Meier analysis and Cox proportional hazard regression were performed. All tests were performed two-tailed and P-values <0.05 were considered significant.

## Results

### Functional characterization of miR-96

**Figure 1:**
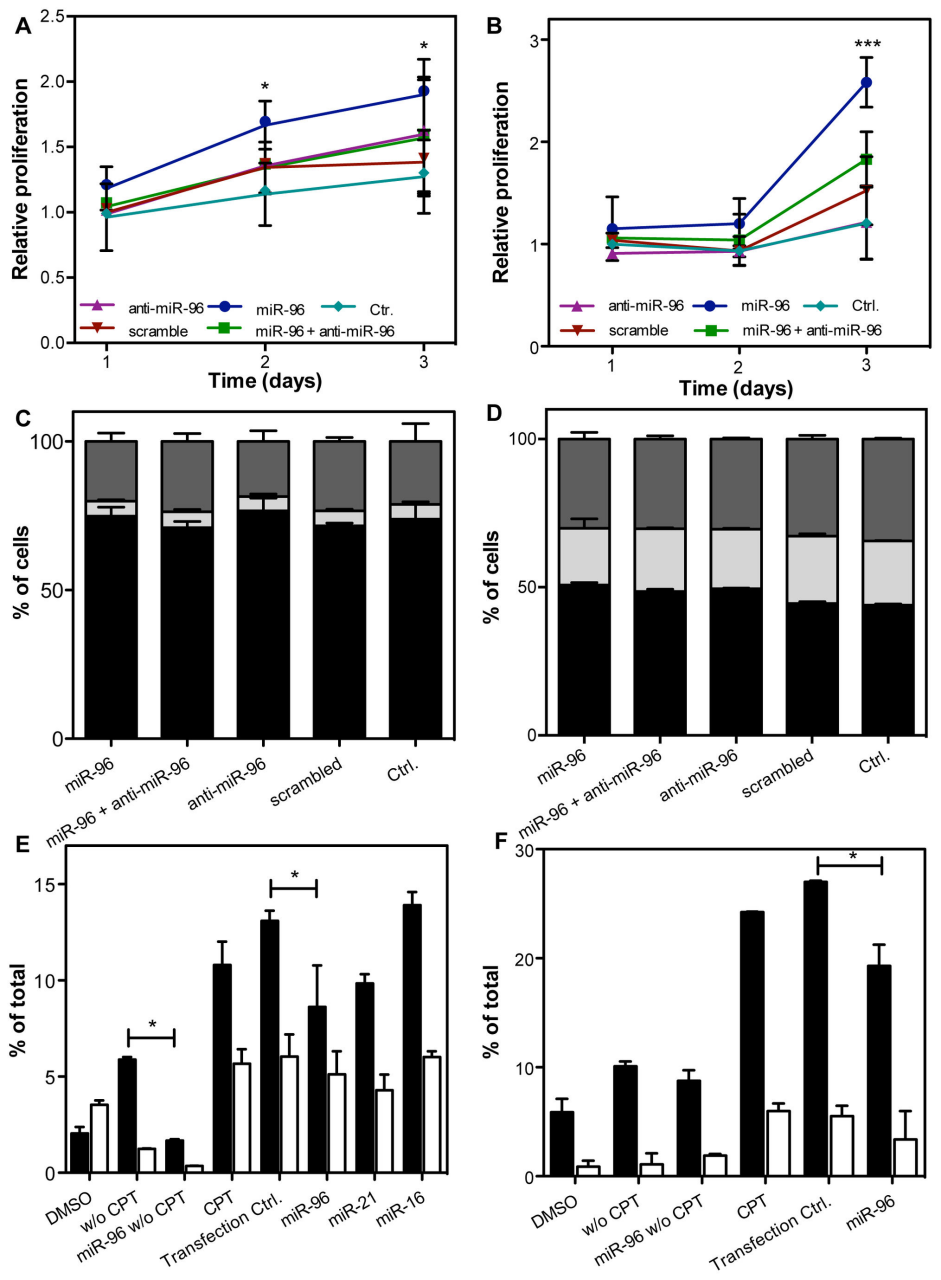
Functional role of miR-96 in vitro. LNCaP and DU145 cells were transfected with 10 nM pre-miR-96, anti-miR-96, pre-miR-NC #1 or combination of pre-miR-96 and anti-miR-96. The effect in (A) LNCaP and (B) DU145 on cell proliferation under serum starvation was measured by MTT assay over three days. All data were normalized on proliferation of control cultures on day 1 and are shown as mean (±SD) of three independent assays. *P <0.05, Two-way ANOVA. Cell cycle transition was measured by PI staining in transfected (C) LNCaP and (D) DU145 cells. Cells were serum starved one day after transfection for another 24 hrs and subsequently fixed and stained. Black, G1-phase; light grey, S-phase; dark grey, G2/M-phase. Data are shown as mean of three independent assays. Apoptosis in CPT-treated (E) LNCaP and (F) DU145 cells. 24 h after transfection cells were treated with 10 µM CPT for 24 h. Cells were stained with Annexin V-FITC and PI. Fraction of early (black bars) and late (white bars) apoptotic cells was measured by flow cytometry. Data are shown as mean (+SD) of three independent assays. *P <0.05; Bonferroni post-test (P < 0.001, Two-way ANOVA).

We have previously reported that miR-96 is upregulated and predicts biochemical relapse in prostate cancer ad therefore hypothesized an oncogenic function of miR-96 in the prostate. The higher expression of miR-96 expression in prostate cancer cells lines in comparison to the benign prostate cell line BPH-1 (Figure S1A) further pointed to an oncogenic function. To establish a functional role of miR-96, LNCaP and DU145 cells were transfected with synthetic miR-96 precursor molecules or the antisense inhibitor. First, we assessed the role of miR-96 on cell proliferation. LNCaP and DU145 cells transfected with miR-96 showed a higher proliferation rate in comparison to the transfection controls (Figure 1A+B). This pro-proliferative effect was partially reversed by cotransfection with its antisense inhibitor.

Next, we addressed the question whether this enhanced proliferation might be due to a release of cell cycle control or decreased apoptosis. To determine the effect of miR-96 on cell cycle regulation, the number of LNCaP and DU145 cells in G1, S or G2/M phase was assessed by flow cytometry upon transfection with miR-96 in serum-starved cells. The majority of LNCaP cells were in G1 under serum starvation (71.0 to 76.7%) and smaller fractions were in S-phase (4.8 to 5.3%) or in G2/M (18.6 to 23.7%, Figure 1C). There were no significant differences (Second-Way ANOVA; P = 0.12) in cell cycle transition in cells transfected with miR-96 precursors and inhibitors in comparison to the controls. DU145 were transitioning faster through cell cycle, resulting in a higher number of cells in G2/M (30.1 to 34.4%) and S-phase (19.2 to 22.7%) and less cells in G1 (43.9 to 50.7, Figure 1D). No significant differences in cell cycle transition were observed upon transfection, thereby affirming the observations in LNCaP cells (Second-way ANOVA, p=0.2). 

As the enhanced proliferation could not be explained by increased cell cycle transition, we investigated the effect of miR-96 transfection on apoptosis. LNCaP and DU145 cells were transfected with pre-miR-96, miR-96 inhibitors or scrambled control and apoptosis was induced with 10 µM camptothecin (CPT). CPT induced apoptosis in LNCaP control cultures with 13.2% early apoptotic cells and 5.6% late apoptotic cells and in DU145 cells (24.2% early and 5.9% late apoptotic cells) (Figure 1E+F). Transient transfection with miR-96 precursors reduced the fraction of early apoptotic and late apoptotic cells by 28% (LNCaP) and 25% (DU145) (Second-way ANOVA, P <0.001). The specificity of the observed effect of miR-96 were confirmed by miR-21, which served as a positive control [22] and exhibited a similar inhibition of apoptosis and miR-16, which served as a negative control and did not affect apoptosis in prostate cancer cells.

We further investigated migration of DU-145 cells upon transfection with miR-96. DU-145 showed a migrative phenotype and were able to remigrate to 60% of the area of the initial wound within 20 h (Figure S2). Transfection with miR-96 precursor or miR-96 inhibitor had no effect on cell migration in comparison to transfection with scrambled control or non-transfected control (Second-Way ANOVA; P = 0.71).

### Identification of miR-96 target genes

**Figure 2:**
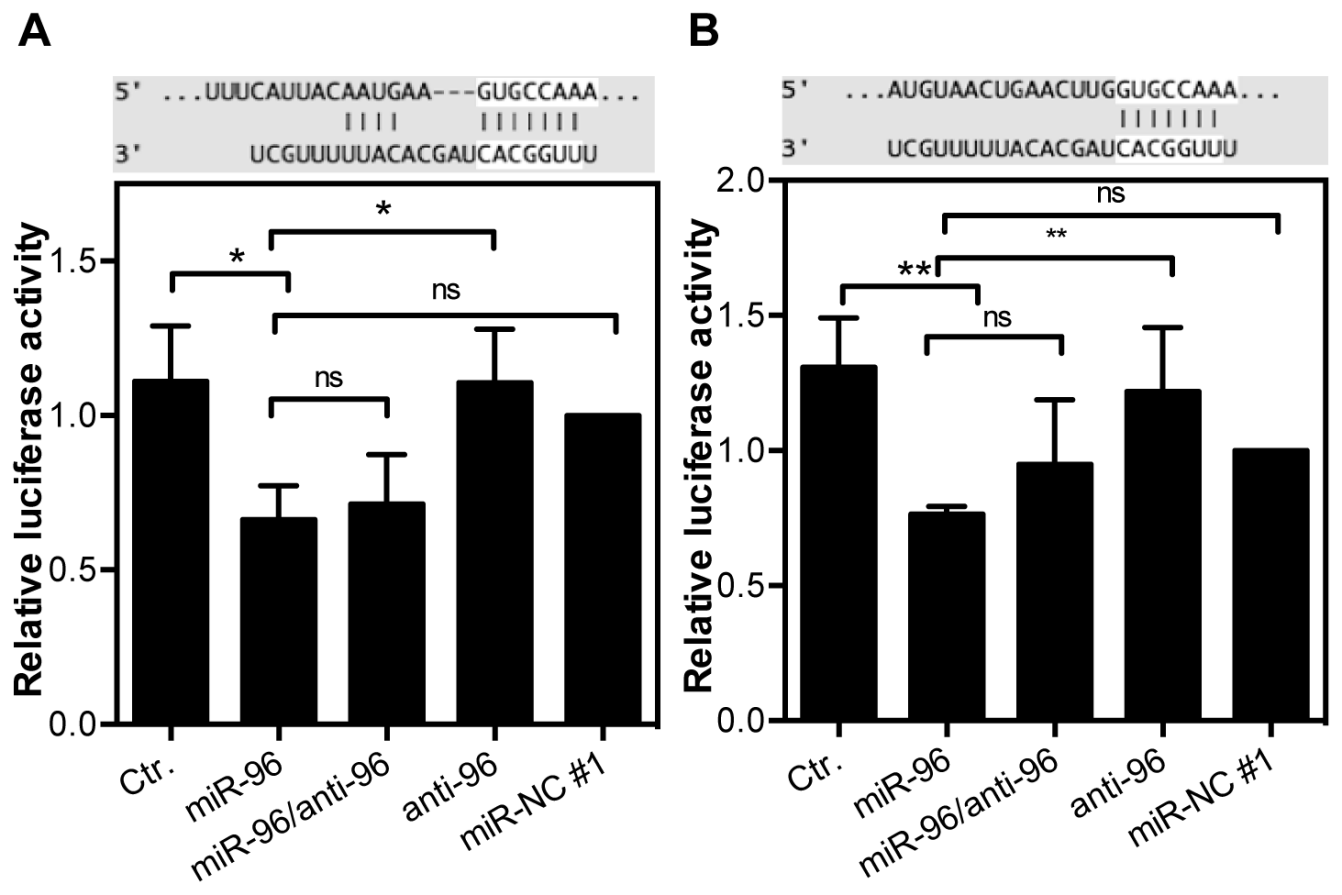
Binding of miR-96 in the 3’ UTR of FOXO1. LNCaP cells were transfected with 10 nM pre-miR-96, anti-miR-96, pre-miR-NC #1 or combination of pre-miR-96 and anti-miR-96 as well as 500 ng pMiR report β-Galactosidase control plasmid and 500 ng pMiR report plasmid for FOXO1 3’ UTR A) binding site 1 at position 264-270 and B) binding site 2 at position 2138-2145. * P <0.05, **P <0.01, One-way ANOVA and Tukey’s multiple comparison post test.

To identify regulated targets, which potentially account for the apoptotic effects, we performed in silico target search in miRecords and regarded the prediction only valid if the target was identified by miRANDA, TargetScan and PicTar, and two additional prediction algorithms. Thus, we identified 209 targets for miR-96 (table S2). The set of predicted target genes was further analyzed regarding their gene ontology (GO) terms to identify apoptosis-related genes. 21 out of 209 predicted miR-96 targets had an apoptosis-related GO term assigned (table S2). We also investigated the number of binding sites and their evolutionary conservation in TargetScan 5.1. and performed a literature search to identify targets with a known tumorsuppressive function in PCa. One of the predicted targets, FOXO1 was chosen for further validation due to its known tumorsuppressive function in prostate cancer. Correlation of miR-96 and FOXO1 transcript expression in LNCaP and DU145 cells shows an inverse correlation of expression (Figure S1A+B). The FOXO1 3’ UTR harbors two 8-mer miR-96 binding sites at position 264-270 and position 2138-2145. While the first binding site is highly conserved, the second miRNA binding site is not. We constructed luciferase-reporters containing 200-300 bp long sequences enclosing the predicted target sites. Cotransfection of both FOXO1 3’ UTR luciferase reporters with pre-miR-96 in LNCaP cells showed a 40% reduction of luciferase activity in comparison to the transfection control (One-way ANOVA, P <0.01), while transfection with the miR-96 inhibitor or scrambled control did not significantly affect luciferase activity (Figure 2A). Addition of the miR-96 antisense sequences released its inhibition only slightly. Although the second miR-96 binding site in the FOXO 3’ UTR is only partially conserved, a similar binding activity with 42% reduction of luciferase activity (ANOVA, P <0.05) was observed in comparison to the control (Figure 2B). Addition of the miR-96 antisense sequence partially diminished this effect, while the antisense sequence alone did not affect luciferase activity. The scrambled miRNA sequence resulted in a small, but no significant inhibition of luciferase activity.

### Reduction of miR-96 target gene expression in vitro

**Figure 3:**
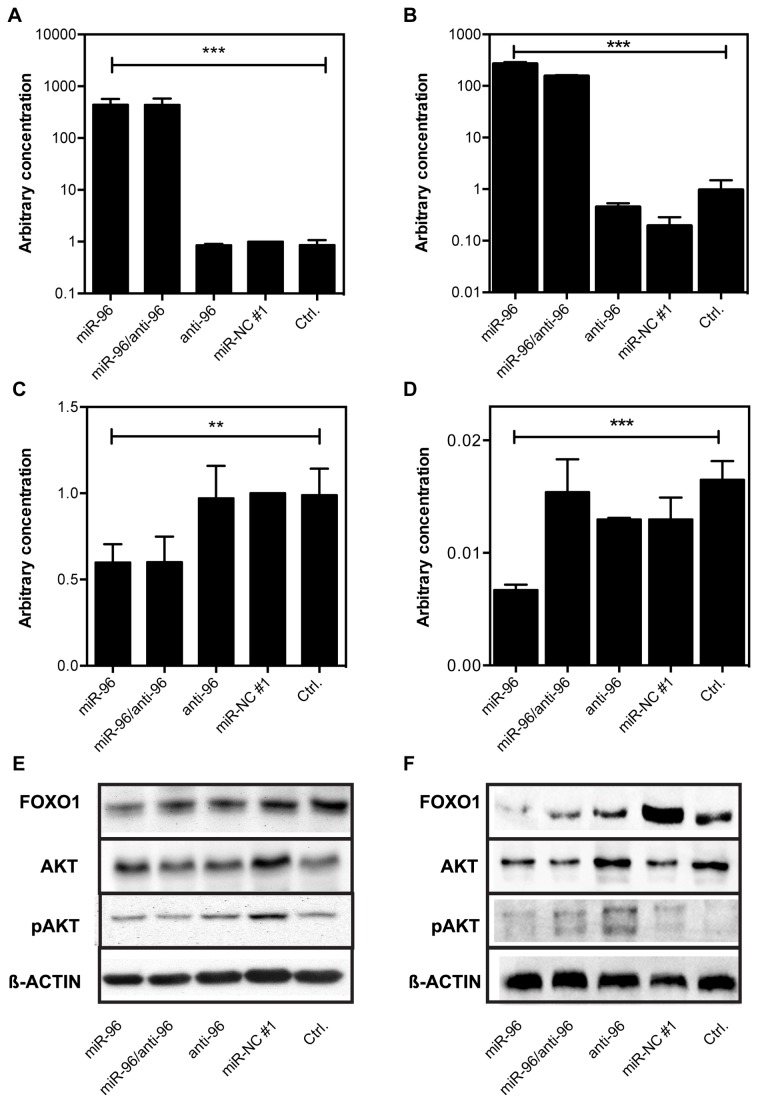
Effect of miR-96 overexpression on FOXO1 expression. Prostate cancer cells were transfected with 10 nM pre-miR-96, anti-miR-96, pre-miR-NC #1 or combination of pre-miR-96 and anti-miR-96. miR-96 expression in (A) LNCaP and (B) DU145 cells and FOXO1 expression in (C) LNCaP and (D) DU145 cells. Data are shown as mean (+SD) of three independent assays. ** P <0.01, *** P <0.001, One-way ANOVA. FOXO1, AKT, and pAKT protein expression in (E) LNCaP and (F) DU145 cells was visualized by western blotting. β-Actin was used as a loading control.

miRNAs are suggested to mainly inhibit protein translation, but some miRNAs have also been shown to lead to an at least partial degradation of the corresponding mRNA [23]. To establish the main mechanism by which miR-96 inhibits FOXO1 in PCa and to prove that the mechanistic binding of miR-96 to the 3-UTR can result in a decreased expression of FOXO1, we transfected LNCaP and DU145 cells with miR-96 precursor and/or the inhibitor. miR-96 levels in both cell lines were 400-times higher in the miR-96 transfected cells and in the cotransfected cells in comparison to the control cultures (ANOVA, P <0.001; Figure 3A+B). Correspondingly, FOXO1 mRNA was reduced more than two-fold in miR-96 transfected cells (ANOVA, P <0.01, Figure 3C+D), while the transfection with the miR-96 antisense sequence and the scrambled miRNA showed no effect on FOXO1 expression levels (Figure 3C+D). Additional to the reduction of the FOXO1 transcript, we observed a 1.6-fold and 2.6-fold reduction of FOXO1 protein in LNCaP and DU145 cells upon ectopic overexpression of miR-96 precursor, while protein levels were not significantly altered in cells transfected with the miR-96 inhibitor or the scrambled control (Figure 3E+F, Figure S3A+B). Taken together, the data support the hypothesis that miR-96 inhibits FOXO1 expression.

FOXO1 activity is directly regulated by phosphorylation by Akt upon external growth signals, resulting in translocalization from the nucleus. To check if miR-96 overexpression significantly alters upstream Akt signaling, we assessed Akt and phosphorylated Akt expression in miR-96 overexpressing cells. Neither Akt nor phosphorylated Akt levels were significantly altered upon transfection with miR-96 precursors or inhibitors (Figure 3E+F), demonstrating that the activity of FOXO1 was not affected by changes in Akt expression or activity. 

**Figure 4:**
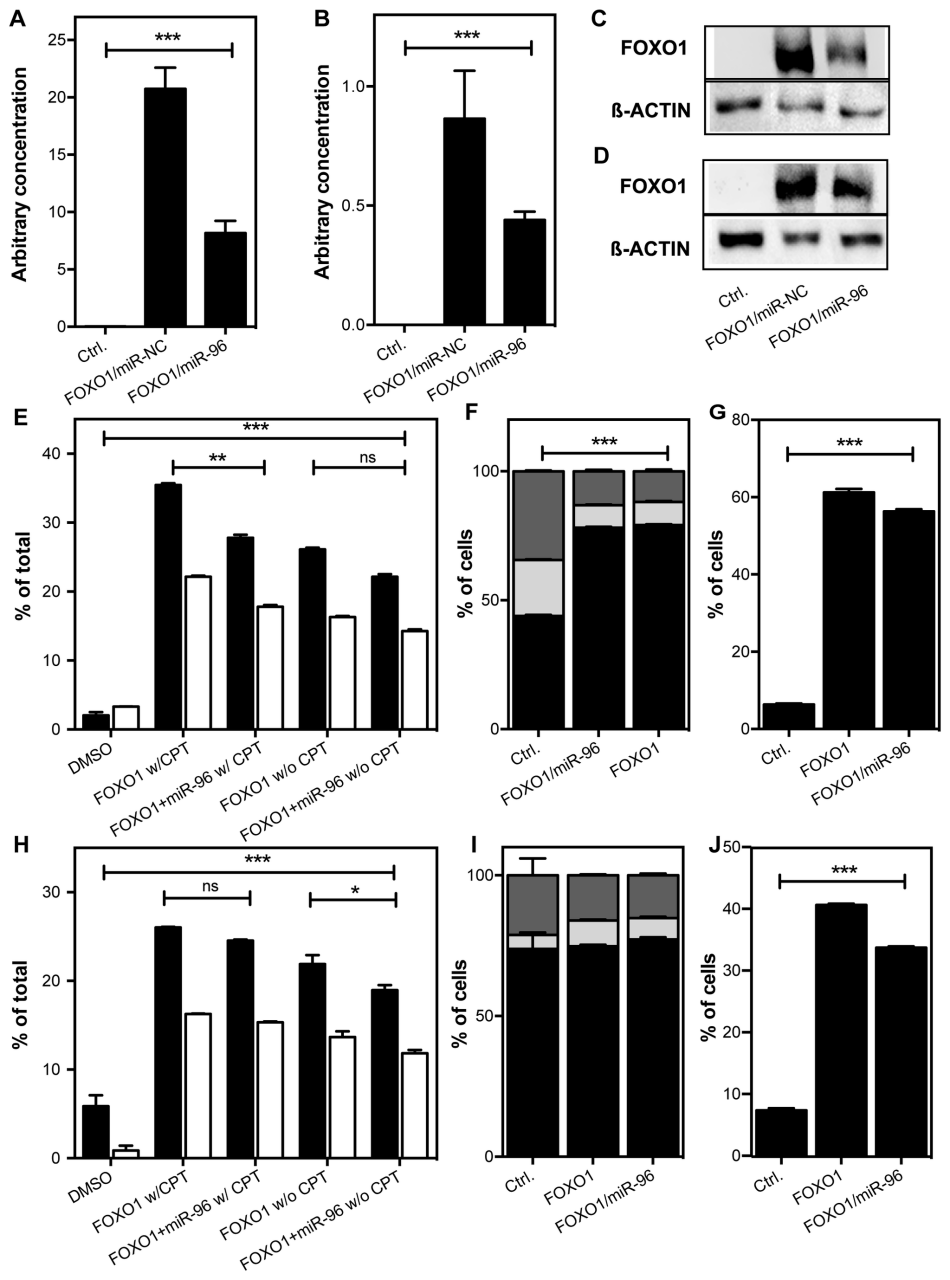
FOXO1 overexpression in prostate cancer cell lines. LNCaP and DU145 cells were transfected with 500 ng FOXO1 full length clone or empty vector, 10 nM pre-miR-96 or pre-miR-NC #1. FOXO1 expression in (A) LNCaP and (B) DU145 cells. Data are shown as mean (+SD) of three independent assays. *** P <0.001, One-way ANOVA. FOXO1 protein expression in (C) LNCaP and (D) DU145 cells was visualized by western blotting. β-Actin was used as a loading control. Apoptosis in CPT-treated (E) LNCaP and (H) DU145 cells. 24 h after transfection cells were treated with 10 µM CPT for 24 h. Cells were stained with Annexin V-FITC and PI. Fraction of early (black bars) and late (white bars) apoptotic cells was measured by flow cytometry. Data are shown as mean (+SD) of three independent assays. ns, P>0.05,*P <0.05,**P<0.01,***P<0.001, Two-way ANOVA, Bonferroni’s post test). Cell cycle transition was measured by PI staining in transfected (F) LNCaP and (I) DU145 cells. Cells were serum starved one day after transfection for another 24 hrs and subsequently fixed and stained. Black, G1-phase; light grey, S-phase; dark grey, G2/M-phase. Data are shown as mean of three independent assays. ***P<0.001, One-way ANOVA. Analysis of subG1 peak in (G) LNCaP and (J) DU145 cells. Data are shown as mean of three independent assays. ***P<0.001, One-way ANOVA.

To further support the hypothesis that miR-96 controls FOXO1 in prostate cancer and thereby protects cells from apoptosis, we transfected LNCaP and DU145 cells with a full length FOXO1 cDNA. Ectopic expression of FOXO1 resulted in a strong upregulation of FOXO1 as validated by RT-PCR and westernblotting in both LNCaP and DU145 cells (Figure 4A-D). Due to the strong staining intensity of FOXO1 in transfected cells, FOXO1 in control cells is not yet visible on the blot and only became visible after longer exposition times. Cotransfection with miR-96 reduced FOXO1 mRNA levels by 2.5- and 1.7-fold in LNCaP and DU145 cells (One-way ANOVA, p<0.001), while protein levels were reduced by 2.8- and 2.1- fold (Figure 4C+D, Figure S3C+D).

In the next step, we assessed, whether FOXO1 has an opposing role to miR-96 in prostate cancer cells and whether the effect might be rescued by cotransfection with miR-96. FOXO1 strongly induced apoptosis in both cell lines (Two-way ANOVA, p < 0.001, Figure 4E+H). Treatment with CPT only had a mild additional effect on the cells. Simultaneous overexpression of miR-96 partially rescued the FOXO1 induced apoptosis in untreated as well as CPT-treated cells, although the effect was small and not significant in all treatments (Figure 4E+H). 

Proliferation was also strongly reduced in FOXO1 transfected LNCaP and DU145 cells, but miR-96 did not significantly reverse the effect (Figure S4A+B). Next we studied the FOXO1 induced effect on cell cycle transition. Expression of FOXO1 induced cell cycle arrest in DU145 but only lead to a small reduction of the G2/M fraction in LNCaP cells (Figure 4F+I). Simultaneous expression of miR-96 had no significant effect on cell cycle transition. Nevertheless, the analysis of the subG1 peak further supported the miR-96 mediated inhibition of FOXO1 induced apoptosis, as the subG1 peak was strongly enhanced by FOXO1 treatment and partially reduced by cotransfection with miR-96 (Figure 4G+J).

In summary, we were able to show that miR-96 can bind to the FOXO1 3’-UTR. Thus miR-96 reduces the total levels of FOXO1 transcript and protein and leads to a mild but consistently observed protection from apoptosis.

### Correlation of miR-96 and FOXO1 expression in human PCa tissue

**Figure 5:**
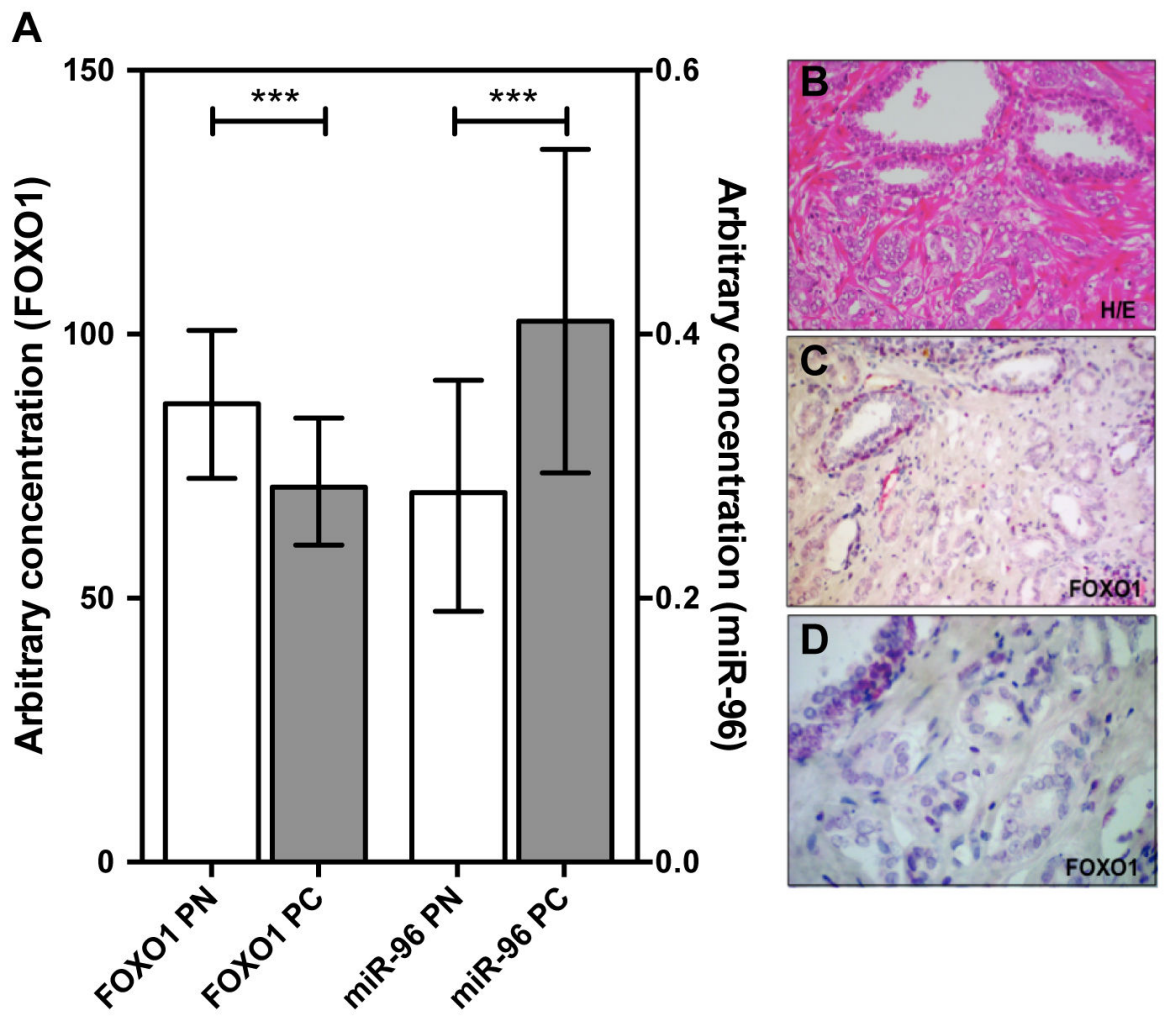
miR-96 expression and FOXO1 expression in PCa specimens. (A) miR-96 and FOXO1 mRNA expression was measured by RT-qPCR in 69 matched PCa (PC) and normal adjacent tissue (PN). All data were normalized to efficiency and interplate control. FOXO1 expression was normalized to the reference gene TUBA1B [19] and miR-96 expression was normalized to miR-130b [6]. Data are shown as median expression (+ interquartile range). FOXO1 expression is displayed on the left y-axis, miR-96 expression on the right y-axis. ***P <0.001; Wilcoxon signed rank test. (B) Haematoxilin-eosin (H/E) staining was performed to identify tumor ducts. (C+D) FOXO1 expression was detected by immunhistochemistry on a tissue microarray containing tissue cores corresponding to 69 PCa specimen. Each core had a diameter of 1.5 mm.

To further establish the role of miR-96 in prostate carcinogenesis, we measured the miR-96 and FOXO1 mRNA expression in 69 PCa specimens with their adjacent normal tissues. Tumor characteristics and clinical-pathological data are summarized in table 1. As described previously [6], miR-96 was significantly upregulated in PCa and was significantly correlated with Gleason score (Figure 5A). In contrast, FOXO1 displayed a median 1.2-fold downregulation in PCa, with 71% of patients having a lower expression of FOXO1 in the tumor (Wilcoxon signed rank test; P <0.001, Figure 5A). FOXO1 expression was able to discriminate between cancer tissue and normal adjacent tissue as assessed by ROC analysis (AUC: 0.70, P >0.001, Figure S5A). The ratio of miR-96 expression in tumor tissue to that in the normal adjacent tissue did not correlate with the corresponding ratio of FOXO1 (r_s_=0.02; P=0.89, Figure S6). 

FOXO1 was not correlated with any clinico-pathological parameter in PCa (data not shown). To determine the prognostic association of FOXO1 mRNA expression in tumor tissue to PCa recurrence indicated by rising values of prostate-specific antigen (PSA) as so-called biochemical relapse after radical prostatectomy, Kaplan-Meier and univariate Cox regression analysis of FOXO1 mRNA expression was performed. FOXO1 expression was normalized to expression in normal adjacent tissue and dichotomized according to the median value. Patients with low FOXO1 transcript expression showed a higher risk of biochemical relapse than patients with high FOXO1 transcript expression (Figure S7A) and ROC analysis showed a borderline significance for discrimination of recurrent and non-recurrent patients by FOXO1 expression (AUC: 0.69, P = 0.05, Figure S5B). We included FOXO1 into our previously established risk model of biochemical relapse [6]. FOXO1 only showed a borderline significance in the multivariate Cox regression model containing the variables Gleason score, miR-96 and FOXO1 mRNA (table S3).

We additionally estimated the protein expression of FOXO1 on a tissue microarray (TMA) comprising of 64 patients (Table 1). Expression of FOXO1 was analysed in normal and in tumor areas determined for each patient. As expression was relatively weak in both tissues it was classified as present or absent. FOXO1 expression was restricted to epithelial cells with a predominant localization in the nucleus in normal tissue and a shift to a cytoplasmatic localization in the tumor cells (Figure 5B-D). FOXO1 expression was significantly reduced in the tumor with expression being detectable in 62% of benign epithelial cells, but only in 31% of tumor cells (Table 2). Since miR-96 expression data were available from 36 patients whose samples were analysed for FOXO1 on the TMA, we compared the expression of both. miR-96 expression was dichotomized according to the median expression. In this subset FOXO1 was expressed in 22% of all cancer specimens, while in the specimen with high miR-96 expression only 15% also had detectable FOXO1 expression. McNemar test revealed a significant inverse relation of miR-96 and FOXO1 protein expression (P = 0.02, table 3).

**Table 2:** Detection of FOXO1 protein in prostate cancer specimen.

	**Tumor**	**Normal**
**FOXO1 positive (%)**	18 (31)	21 (62)
**FOXO1 negative (%)**	40 (69)	13 (38)
**Analyzed specimen (% of all)^a^**	58 (91)	34 (53)

^a,^ total number of patients on the TMA was 64. Within these FOXO1 detection could be analyzed in 58 cancer specimen, while normal tissue was only traceable in 34 of the spots.

**Table 3:** Association of miR-96 and FOXO1 expression in prostate cancer specimens.

		**FOXO1**		P = 0.02*
		**0^a^**	**1^a^**	
**miR-96**	**0^b^**	11	5	16 (44%)
	**1^b^**	17	3	20 (56%)
		28 (78%)	8 (22%)	36

a 0, not expressed; 1, expressed.

b 0, expression < median expression; 1, expression ≥ median expression.

* McNemar test.

## Discussion

In this study, we investigated the functional role of miR-96 in PCa. The miR-96 cluster is upregulated in PCa in comparison to normal adjacent tissue and is associated with biochemical relapse [6]. We show that miR-96 promotes proliferation and inhibits apoptosis in PCa cells lines, an effect that can be partially explained by its ability to regulate the pro-apoptotic transcription factor FOXO1.

miR-96 controls circadian rhythm via ADCY6 [24,25], and is associated with progressive hearing loss [26]. Overexpression of the miR-96 cluster has also been associated with other cancer types [27–31]. Few studies investigated the function of miR-96 in cancer so far, but mainly confirmed an oncogenic role of this miRNA. In the prostate the mir-183-96-182 cluster controls zinc homeostasis [32]. In breast cancer, miR-96 regulates proliferation, anchorage-independent growth and transition of cells from G1 to S-phase [28]. In endometrial carcinoma, the effect on cell cycle has been confirmed and its impairment of apoptosis has been described [15] and miR-96 controls invasion and differentiation in bladder cancer cell lines via regulation of IRS1 and MAP4K1 [33]. Only in pancreatic cancer, miR-96 was described to have tumorsuppressive properties by downregulating KRAS thereby impairing cancer cell invasion and migration as well as tumor growth in vivo [34].

Having revealed a function of miR-96 in inhibition of apoptosis in PCa, putative targets of miR-96 mediated inhibition were identified and the direct regulation of FOXO1 validated. FOXO1 is a downstream mediator of CPT-triggered apoptosis [35–37]. Upon DNA damage mediated by CPT, phosphorylation of FOXO1 at serine-249 by CDK2 is inhibited, thus resulting in translocalisation of FOXO1 to the nucleus [36]. FOXO1 activity is also regulated by phosphorylation by CDK1 [38] and AKT [13], acetylation/deacetylation [39] and ubiquitination [40]. In a non-phosphorylated state, FOXO1 transcriptionally regulates expression of genes involved in cell cycle, as *CDKN1B* [11] and *RBL2* [41] or proapoptotic genes as *BAX* [12] and *TRAIL* [42]. 

FOXO1 is silenced in PCa by several mechanisms. As mentioned before, FOXO1 activity is inhibited because of hyperactive Akt signaling, which occurs in up to 50% of PCa and is mostly a result of PTEN deletion [43]. Further the *FOXO1* locus at 13q14 can be deleted in PCa patients [44]. We provide an alternate mechanism by which FOXO1 is regulated, namely by binding of miR-96 to two sites in its 3’ UTR. Regulation of FOXO1 by miR-96 was confirmed previously in breast cancer, classical Hodgkin lymphoma as well as in endometrial carcinomas [14–16,45]. FOXO3A, another member of the forkhead box O family of transcription factors is also regulated by miR-96 expression in breast cancer, which results in subsequent downregulation of CDKN1B and CDKN1A [28]. 

This study contradicts earlier studies regarding the specific function of miR-96 in cancer. In contrast to other, we did not observe control of cell cycle transition or regulation of cell motility and invasion. Reason for these discrepancies might be found in technical differences or cell type-dependent differences. Albeit the observation that FOXO1 overexpression results in cell cycle arrest in DU145 cells, we could not observe an opposing effect or a rescue by miR-96 on cell cycle. Yet analysis of the subG1 confirmed the results from Annexin V staining and thus ruled out that technical errors obscured the results. Yet, we cannot rule out that the rather mild inhibition of FOXO1 by miR-96 by only 50% is not sufficient to induce cell cycle arrest. Beside the technical pitfalls, it is probable that miR-96 targets several other pro-apoptotic molecules beside FOXO1 in prostate cancer, which results in a predominant inhibition of apoptosis. The in-silico target prediction identified 20 other pro-apoptotic molecules as putative miR-96 targets, for example ITPR1, PRKCE, PPP3R1 and TP53INP1. We analysed binding of miR-96 to ITPR1 and could not see significant binding (data not shown), but have not studied regulation of other pro-apoptotic molecules so far. Taken together, although technical pitfalls exist, effects of miR-96 might be tissue and context dependent. This hypothesis might be supported by the fact that miR-96 acts as a tumorsuppressor in pancreatic cancer [34], although all other studies reported an oncogenic role of this miRNA, but needs to be addressed and verified in future studies.

A drawback in this study is the failed inhibition of miR-96 by the antisense inhibitor. We were not able to see a consistent effect of anti-miR-96 transfection on miR-96 levels or FOXO1 expression or in functional studies. In some assays, anti-miR-96 seemed to partially rescue the miR-96-dependent effects but this was also inconsistent. Thus we have to assume that the inhibition strategy was insufficient. In future studies, we propose to repeat the experiments with a different transfection strategy, for example by using LNA directed against miR-96 that have been shown to effectively relieve target gene repression in classical Hodgkin lymphoma cells [16].

In conclusion, our data support the hypothesis that the regulation of apoptosis by miR-96 is dependent on its regulation of forkhead transcription factors, although there seem to be cell line and tissue specific differences in the functional output.

To confirm that the inhibition of FOXO1 by miR-96 in PCa is of relevance in vivo, the expression of miR-96 was correlated with FOXO1 transcript and protein expression in matched primary PCa and normal adjacent tissues. The observation that miR-96 expression is only inversely related to FOXO1 protein expression, but not mRNA expression suggests a predominantly translational inhibition in vivo. Also an association of low FOXO1 with biochemical relapse in PCa was observed. Cytoplasmic phosphorylated FOXO1 has been associated with biochemical relapse before [46]. Due to the small sample size and generally low clinical failure rates in prostate cancer [47] use of cancer-specific or overall survival as an endpoint in the survival analysis was not feasible, thus limiting the results. Yet, PSA recurrence is a sensitive and so far the earliest markers for recurrent prostate cancer, making it a valid endpoint for survival analysis.

In summary, the characterization of miR-96 identified a novel inhibitor of apoptosis in PCa. The anti-apoptotic function of miR-96 is in part to be explained by its inhibition of FOXO1. Yet, miR-96 most certainly targets an abundance of other targets. To identify those targets, high-throughput profiling, especially of altered protein expression might be a helpful tool.

## Supporting Information

Figure S1 **miR-96 and FOXO1 expression in prostate cancer cell lines.**
(TIF)

Figure S2 **Effect of miR-96 on migration.**
(TIF)

Figure S3 **Quantification of FOXO1 staining intensity.**
(TIF)

Figure S4 **Proliferation in FOXO1 transfected cells.**
(TIF)

Figure S5 **ROC Analysis of FOXO1 expression in PCa.**
(TIF)

Figure S6 **Correlation of miR-96 and FOXO1 in prostate cancer specimen.**
(TIF)

Figure S7 **Analysis of recurrence free survival.**
(TIF)

Table S1 **Overview of PCR primers and their application.**
(RTF)

Table S2 **Predicted miR-96 targets and their GO terms.**
(XLS)

Table S3 **Multivariate Cox regression.**
(RTF)
