# Supplementary figures and images for: The Antiapoptotic Function of miR-96 in Prostate Cancer by Inhibition of FOXO1
Source: PLoS One. 2013 Nov 19;8(11):e80807. doi: 10.1371/journal.pone.0080807 (PMC3834337; doi:10.1371/journal.pone.0080807)

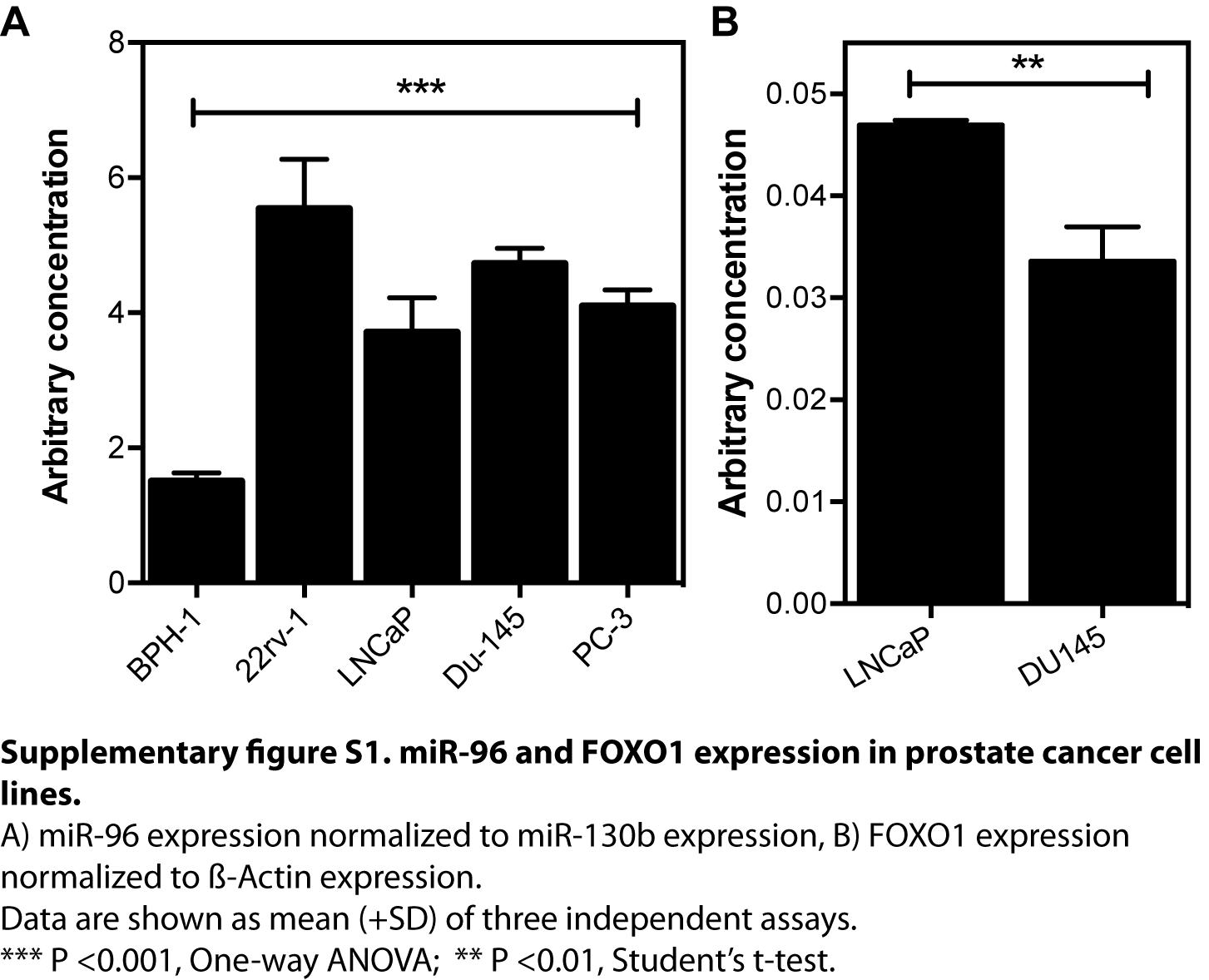

Supplement: Figure S1 — miR-96 and FOXO1 expression in prostate cancer cell lines. (TIF) [file pone.0080807.s001.tif]

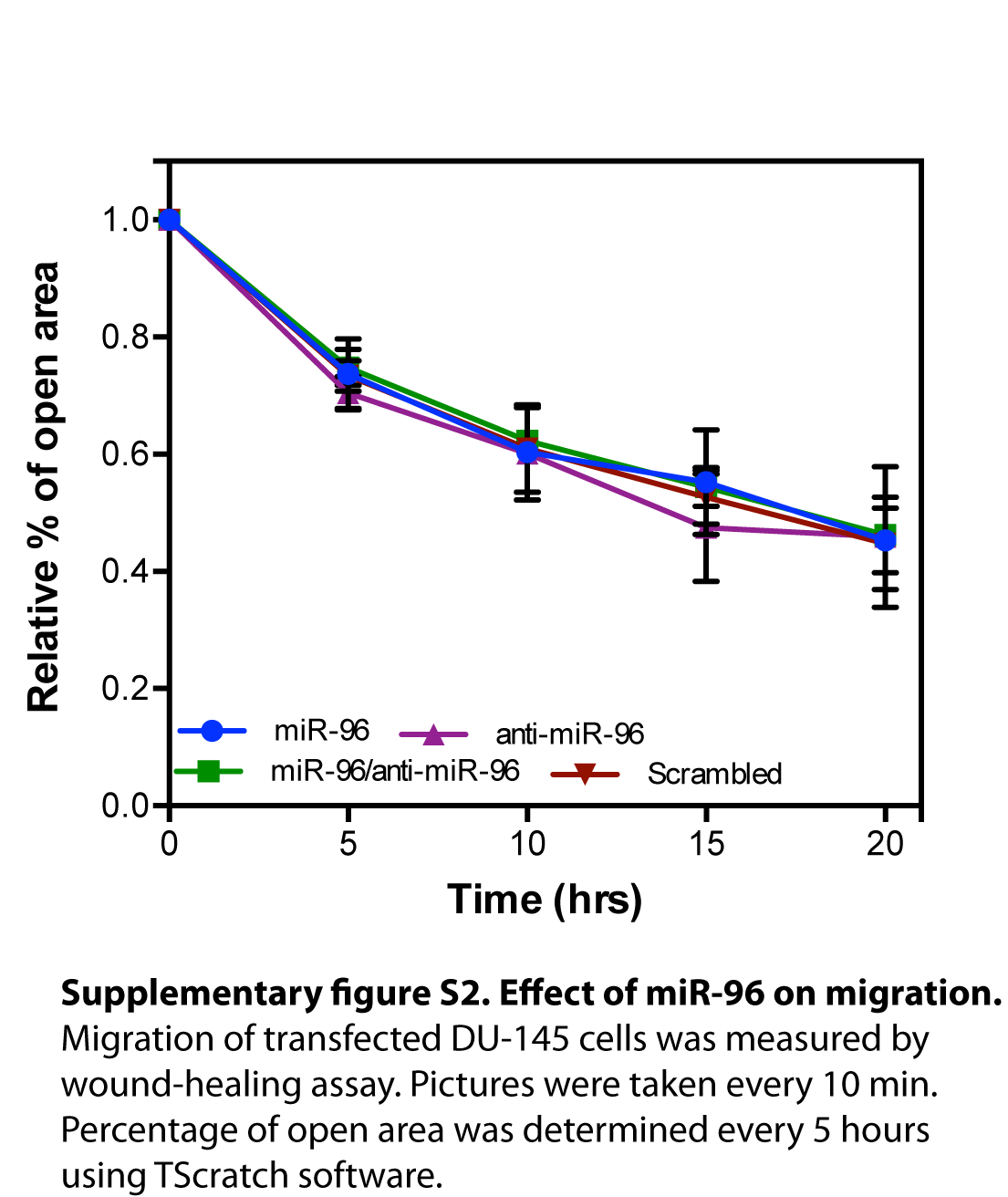

Supplement: Figure S2 — Effect of miR-96 on migration. (TIF) [file pone.0080807.s002.tif]

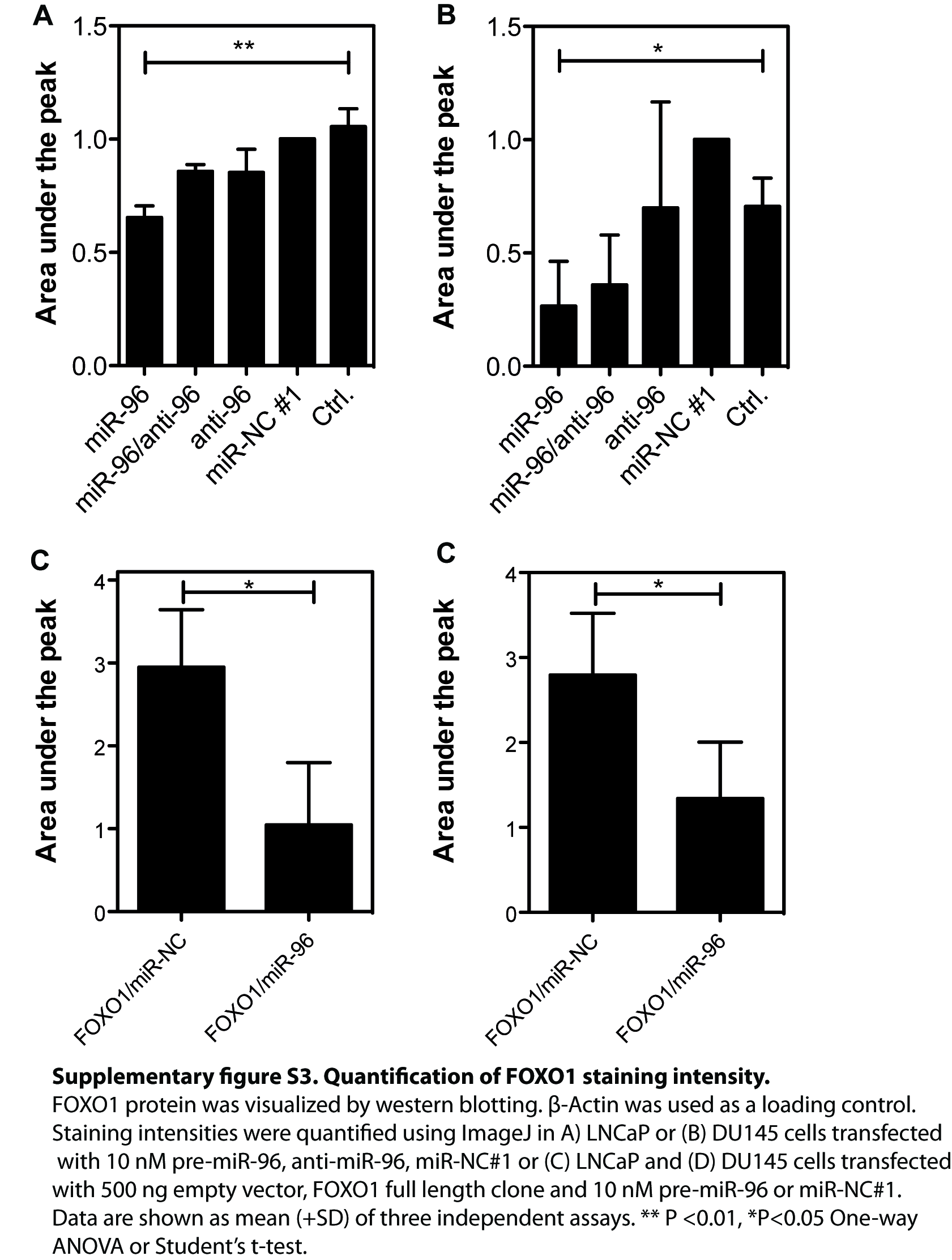

Supplement: Figure S3 — Quantification of FOXO1 staining intensity. (TIF) [file pone.0080807.s003.tif]

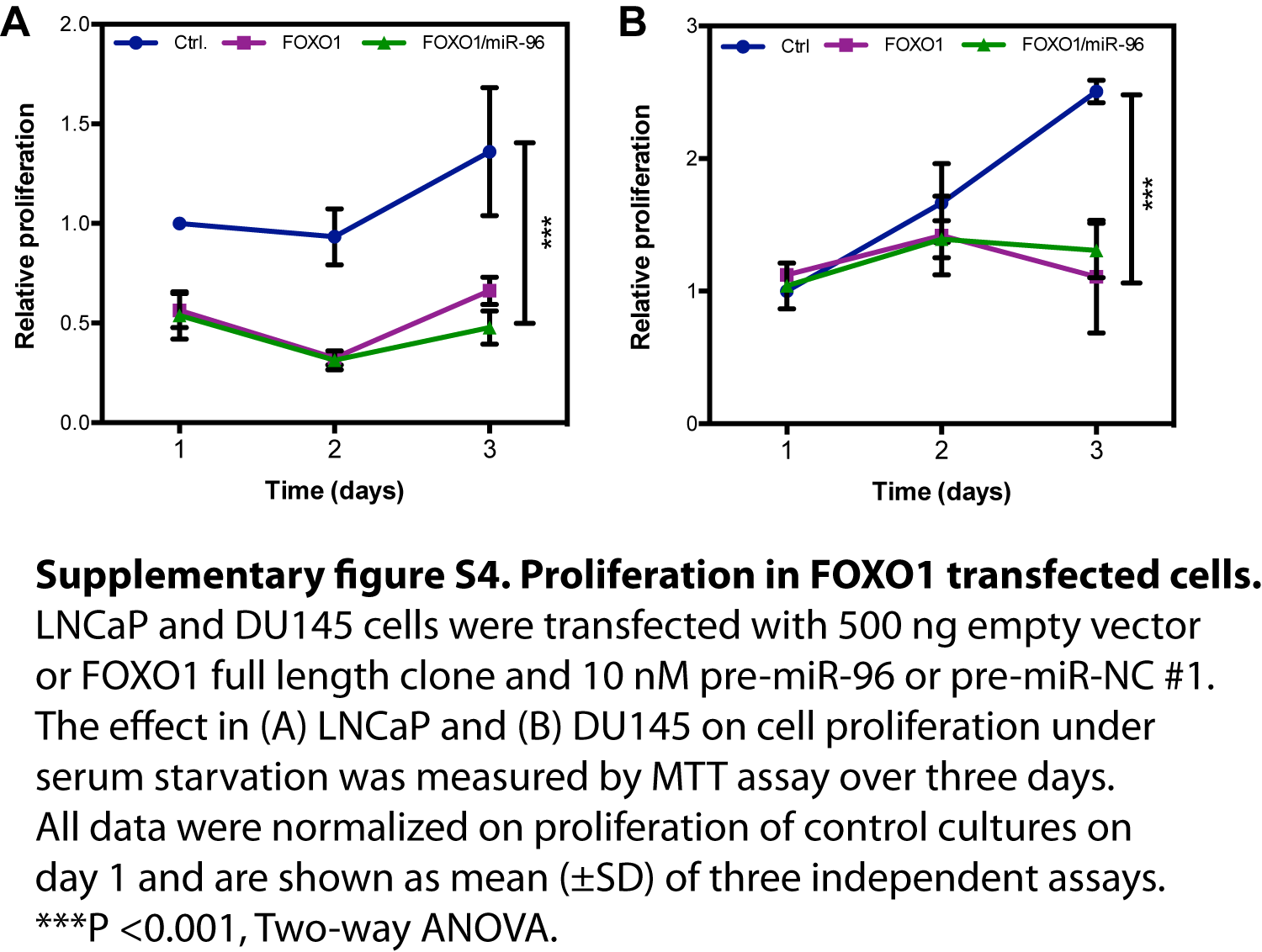

Supplement: Figure S4 — Proliferation in FOXO1 transfected cells. (TIF) [file pone.0080807.s004.tif]

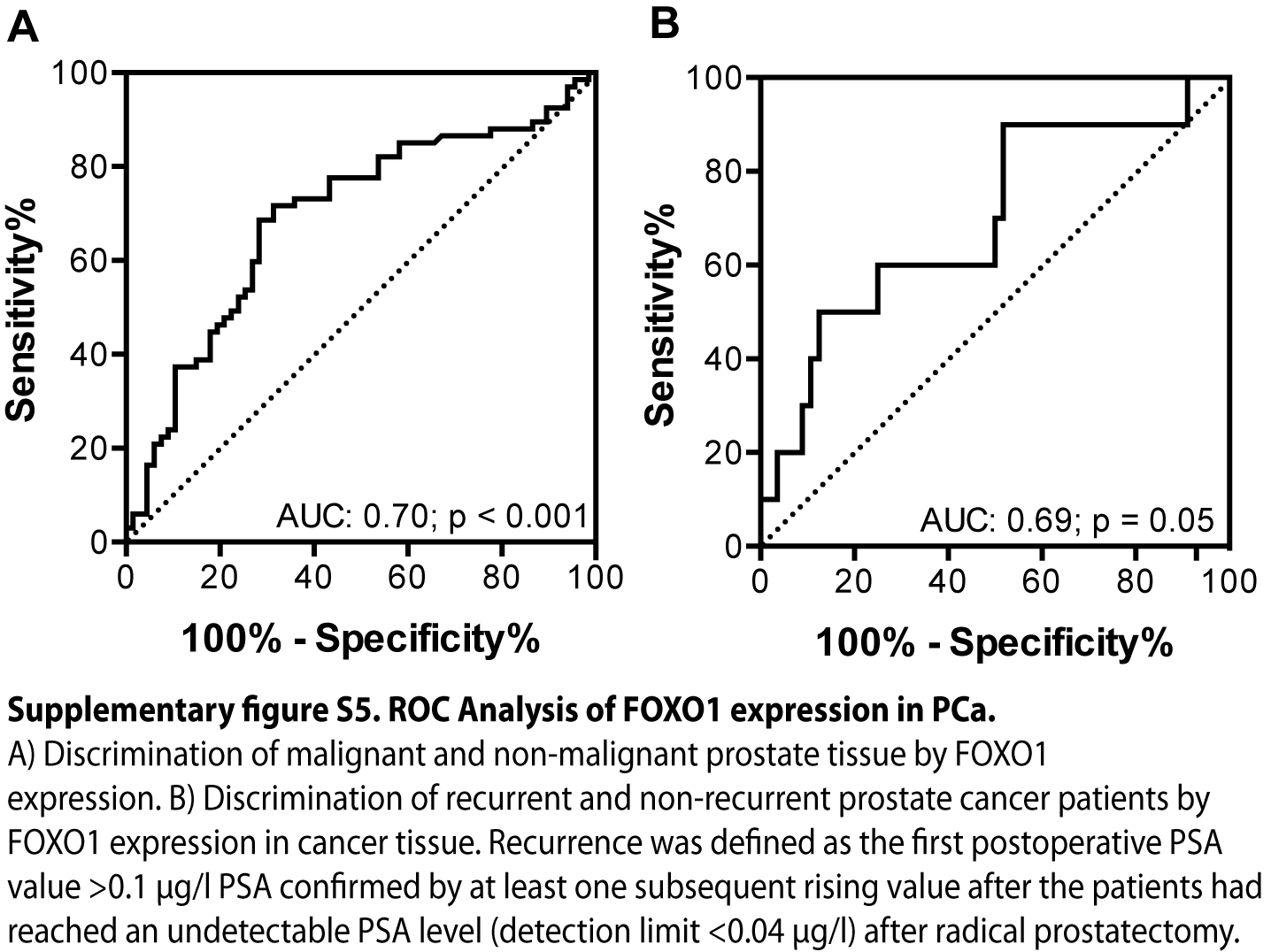

Supplement: Figure S5 — ROC Analysis of FOXO1 expression in PCa. (TIF) [file pone.0080807.s005.tif]

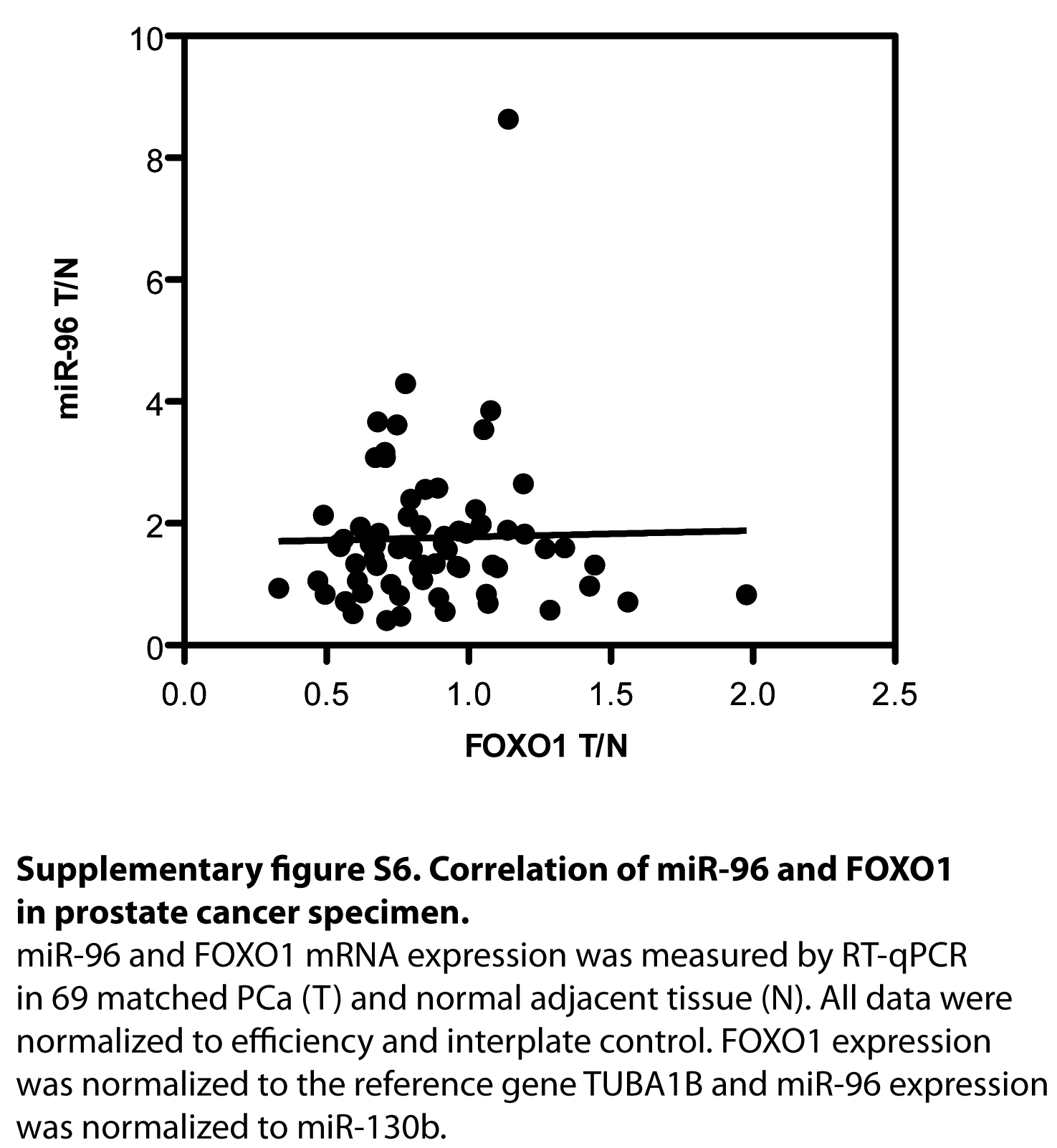

Supplement: Figure S6 — Correlation of miR-96 and FOXO1 in prostate cancer specimen. (TIF) [file pone.0080807.s006.tif]

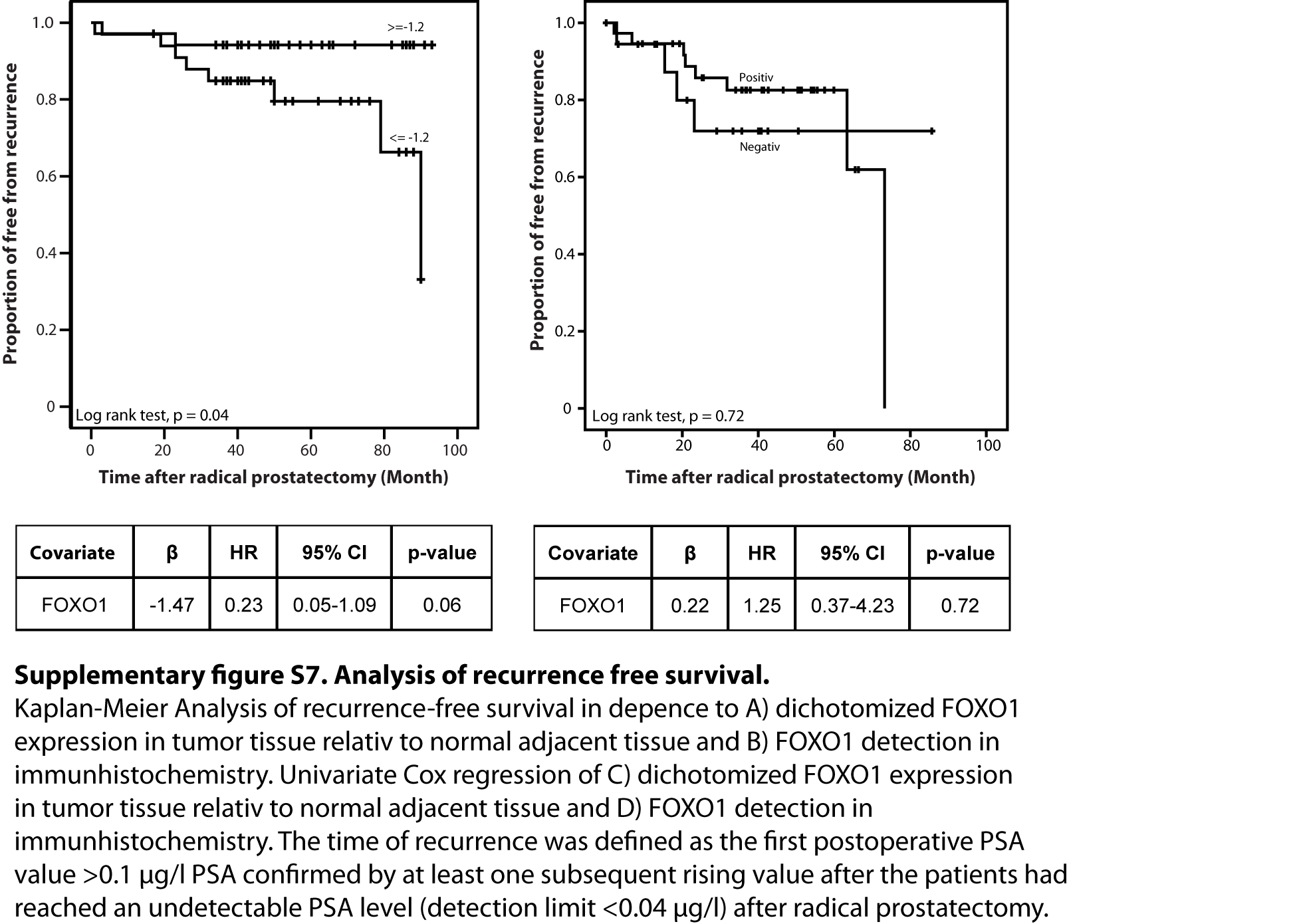

Supplement: Figure S7 — Analysis of recurrence free survival. (TIF) [file pone.0080807.s007.tif]
