# Supplementary material for: The Antiapoptotic Function of miR-96 in Prostate Cancer by Inhibition of FOXO1
Source: PLoS One. 2013 Nov 19;8(11):e80807. doi: 10.1371/journal.pone.0080807 (PMC3834337; doi:10.1371/journal.pone.0080807)
Supplement: Table S1 — Overview of PCR primers and their application. (RTF) [file pone.0080807.s008.rtf]

Supplementary table S1. Overview of PCR primers and their application.

Name	Application	UPL probe	Sequence (5'  3')	Product size	
FOXO1_SYBR_fw	SYBR green qPCR		AAGAGCGTGCCCTACTTCAA	232	
FOXO1_SYBR_rv	SYBR green qPCR		GGCTTCGGCTCTTAGCAAAT		
TUBA1B_SYBR_fw	SYBR green qPCR		CCAGATGCCAAGTGACAAGA	241	
TUBA1B_SYBR_rv	SYBR green qPCR		GATCTCCTTGCCAATGGTGT		
FOXO1_UPL_fw	UPL qPCR	#11	AAGGGTGACAGCAACAGCTC	86	
FOXO1_UPL_rv	UPL qPCR		TTCTGGCACACGAATGAACTTG		
TUBA1B_UPL_fw	UPL qPCR	#64	CCTTCGCCTCCTAATCCCTA	87	
TUBA1B_UPL_rv	UPL qPCR		AGCAGGCATTGCCAATCT		
FOXO1_bd1_fw	PCR (Cloning)		GACTAGTCTTTCGTCAGACTTGGCAGC	301	
FOXO1_bd1_rv	PCR (Cloning)		GGAGCTCAAGACATGAGGCCCATCACA		
FOXO_bd2_fw	PCR (Cloning)		GACTAGTATCATCCTCATTTTGGGGC	201	
FOXO1_bd2_rv	PCR (Cloning)		GGAGCTCTAATGAACAAATGGGGGCTC		
Underlined sequences indicate the introduced SacI and SpeIII recognition sites; UPL: Universal probe library.
