# Supplementary material for: The Antiapoptotic Function of miR-96 in Prostate Cancer by Inhibition of FOXO1
Source: PLoS One. 2013 Nov 19;8(11):e80807. doi: 10.1371/journal.pone.0080807 (PMC3834337; doi:10.1371/journal.pone.0080807)
Supplement: Table S3 — Multivariate Cox regression. (RTF) [file pone.0080807.s010.rtf]

Supplementary table S3. Multivariate Cox regression.Covariate	β	R	95% CI	p-value	
FOXO1	-1.38	0.25	0.05-1.21	0.08	
miR-96	1.87	6.48	1.09-38.59	0.04	
Gleason score	1.14	3.11	1.16-8.34	0.02	
